# Key considerations to inform operational EU‐specific protection goals: An example for non‐target terrestrial plants

**DOI:** 10.1002/ieam.4420

**Published:** 2021-06-14

**Authors:** Christian Bogen, Christoph Julian Mayer, Joanna Davies, Virginie Ducrot

**Affiliations:** ^1^ Bayer AG Monheim Germany; ^2^ BASF SE Limburgerhof Germany; ^3^ Syngenta, Jealott's Hill International Research Centre Berkshire UK

**Keywords:** Biodiversity, Ecosystem services, Non‐target plants, Protection goals, Trade‐offs

## Abstract

This paper complements recent considerations of specific protection goals (SPG) to inform risk assessments for non‐target terrestrial plants (NTTP) in the European Union. The SPG options in‐field appear to be of the most disruptive potential from agronomic perspective and are therefore investigated in more detail. Overarching prerequisites have been identified that need to be accounted for to ensure that any of the potential SPG options remain operational in a sustainable agricultural context. As soon as crop production is considered a desired ecosystem service for the in‐field, its specific requirements in the context of sustainable agriculture have to be factored in. Good agricultural practices (GAPs), potential ecosystem disservices (e.g. weeds, pests and diseases) and supporting and regulating services need to be considered to ensure a successful and sustainable delivery of the ecosystem service crop production. Concerning in‐field SPG options for NTTP specifically GAPs related to integrated weed management (IWM) require detailed assessment, as they individually and in combination have the purpose of weed control. Therefore, they result in specific implications to the environment, ecosystem services and biodiversity within the context of sustainable agricultural production. When diverging in‐field ecosystem services are considered for the same context, the protection goals options require an additional assessment of synergies and trade‐offs between the relevant ecosystem services (e.g. crop production, climate regulation and aesthetic values), a corresponding weighing and prioritization. Similarly, for biodiversity conservation, the trade‐offs and synergies between sustainable crop production and specific habitat requirements need to be accounted for. Consequently, an interdisciplinary approach can ensure that SPG are operational by integrating a broad understanding of cropping systems, the environmental impact of the tools a farmer uses and the link between habitat availability, the impact of any of the applied tools on habitat quality and the broader landscape context. *Integr Environ Assess Manag* 2021;17:905–910. © 2021 Bayer AG, BASF SE and Syngenta. *Integrated Environmental Assessment and Management* published by Wiley Periodicals LLC on behalf of Society of Environmental Toxicology & Chemistry (SETAC).

## KEY POINTS

- An interdisciplinary approach can ensure that specific protection goals for environmental risk assessments for crop protection products are operational and realistic in a sustainable agricultural context.
- Tools and management strategies used within sustainable agriculture and the natural environment interact with each other and can exert both positive and negative effects, respectively, thus requiring a holistic approach for setting specific protection goals.
- In particular, the sustainable delivery of the ecosystem service “crop production” comes with agronomical and environmental constraints that both need to be accounted for when defining in‐field specific protection goals for crop protection.
- Ecosystem services delivery and biodiversity conservation also have inherent trade‐offs and synergies that need to be considered and prioritized within the derivation of specific protection goals.

## BACKGROUND AND OBJECTIVES

Environmental safety is one of the key concern when it comes to the application of chemical crop protection products. Therefore, it is mandatory to demonstrate safe use to be able to register those products. For this purpose, Regulation (EC) No. 1107/2009 (2009) was established in the European Union to balance safeguarding the competitiveness of community agriculture with an appropriately high level of protection for humans, animals, and the environment. Specific protection goals (SPGs) are defined to ensure that pesticide use is acceptable for the environment. Once defined, these protection goals become the benchmark of risk assessment and corresponding guidance documents (EFSA, 2016).

For non‐target terrestrial plants (NTTP), EFSA published a scientific opinion on the risk assessment, which reviewed literature concerning the ecotoxicology of NTTP (EFSA, 2014). It also included proposals for potential definitions of protection goals for off‐field, in‐field, and endangered plant species. The protection goals were proposed by taking into account biodiversity and ecosystem services and so reflect the main ideas of the EFSA guidance on protection goals published in 2016 (EFSA, 2016).

In addition, the scientific opinion proposed a redefinition of the term NTTP. In contrast to the currently applicable guidance where all non‐crop plants located outside the treatment area are considered NTTP (EC, 2002), the proposed redefinition also includes “those [plants] growing within fields that are not the intended pesticide target” (EFSA, 2014).

In 2017, Arts et al. revisited EFSA's 2014 proposal for NTTP protection goals and explored the protection goal options from a Dutch perspective with the aim of facilitating discussions at EU level. For this purpose, Arts et al. (2017) evaluated options for in‐field and off‐field protection goals, using the redefinition of NTTP as proposed by EFSA. For the in‐field situation, Arts et al. considered three protection goal options, namely the “maximal weed reduction option,” the “moderate weed reduction option”, and the “beneficial weed protection option” and their ecological consequences. This paper intends to complement their review by highlighting two overarching prerequisites that are directly related to ecosystem services: biodiversity and sustainable agriculture. They require consideration to ensure that the protection goals are operational in a sustainable agricultural context that conserves both land and water, is environmentally non‐degrading (especially focusing on soils), technically appropriate, economically viable, and socially acceptable (FAO, 2014). The objective of this communication is to ensure that SPGs for plant protection products are consistent with the principles of sustainable farming and therefore suggests a holistic approach encompassing the different tools that are used in good agricultural practices (GAPs).

## THE REQUIREMENTS OF THE ECOSYTEM SERVICE CROP PRODUCTION

The EFSA Scientific Opinion (2014) followed the approach of the Millennium Ecosystem Assessment (2005) and listed numerous ecosystem services that were considered relevant to the in‐crop and off‐crop. In the scientific opinion, food, fiber, and fuel were initially acknowledged as provisioning services. However, the agronomic requirements and environmental implications were not further investigated. Although other ecosystem services and biodiversity aspects were considered in detail in order to inform protection goals, the omission of the agronomic requirements to safeguard the provision of the ecosystem service crop production constitutes a major data gap for the assessment.

This data gap might be acceptable if crop production is not considered a relevant ecosystem service for the in‐field (e.g., in the case of farming for the purpose of nature conservation or to create an aesthetic landscape for tourism). However, as soon as crop production is considered a desired ecosystem service, its specific requirements in the context of sustainable agriculture have to be factored in to ensure that an in‐field protection goal remains operational from a sustainable agricultural perspective.

Crops are considered a subcategory the service food production (Millennium Ecosystem Assessment, 2005). The successful provision of this service may rely partially on regulating services (e.g., water regulation, erosion regulation, pest regulation and pollination) but simultaneously requires human intervention, for example via agricultural practices. When the context of sustainable agriculture is considered, there are overlaps with specific ecosystem services (e.g., conservation of land and water) and environmental targets (e.g., conservation of plant and animal genetic resources), but additionally elements that refer to technical relevance, economic viability, and social acceptability (FAO, 2014).

GAPs play a decisive role in sustainable agriculture. Though there are varying approaches to GAPs, they can usually be considered a process based on the management choices by farmers with the aim of improving the sustainability of agriculture (FAO, 2007). According to FAO (2007), there are several common principles. In crop production, GAPs maximize the biological benefits of weed control by considering a wide array of weed control measures (competition, mechanical, biological, and herbicide options). All of these measures can have direct consequences for non‐crop plant populations in‐field. As GAPs consider the process, where integrated management practices may be used within (FAO, 2007), this would also generally include integrated weed management (IWM). While there might still be discord about the exact definition of IWM, it encompasses conceptually the inclusion of a range of different technologies with a corresponding suite of tactics (Owen et al., 2014).

In general, weed control measures can be considered a relevant contribution to safeguarding the ecosystem service crop production, because non‐crop plants can cause an overall 34% yield loss owing to competition with crop plants for inorganic resources (Oerke, 2006). Compared with animal pests and pathogens with potential yield reductions of 18% and 16%, respectively, weeds constitute a major ecosystem disservice to crop production (Oerke, 2006).

Indeed, there are substantial concerns about the general presence of weed populations in‐field (Norsworthy et al., 2012), which lead to the advice of a wide array of best management practices and recommendations from an agronomic perspective. These range from an in‐depth understanding of the biology of the weeds present in the field, a reduction in the number of seeds in the soil seedbank, weed‐free seed material, routine scouting to using multiple modes of action for herbicide applications (e.g., acetolactate synthase‐inhibiting and protoporphyrinogen oxidase‐inhibiting herbicides), taking advantage of crop competitiveness to suppress weeds, and the use of appropriate mechanical and biological management practices (Norsworthy et al., 2012).

Nonetheless, there have been suggestions in the literature that a certain amount of weed be tolerated, for example, in form of thresholds and linked to a satisfactory or the necessary extent of control (Barzman et al., 2015; Bürger et al., 2008; Keller et al., 2014). These considerations could relate to the “moderate weed reduction option” and the “beneficial weed protection option” as described by Arts et al. (2017) and the reasoning expressed in the EFSA Scientific Opinion (2014). However, agronomic thresholds for harmful weeds can vary greatly depending on crop and weed species (Swanton et al., 1999). For example, for winter wheat, 20–30 plants/m^2^ can be tolerated in the case of grass weeds, whereas only 0.1–0.5 plants/m^2^ of *Galium aparine* are considered acceptable before reaching the threshold (Gerowitt & Heitefuss, 1990). In general, low threshold levels are usually suggested for weeds that are difficult to control. Apart from that, weeds often consist as a community of multiple species, exhibit patchy distribution, and can become persistent in the seed bank, making the concept of economic thresholds for weeds challenging in practice (Barzman et al., 2015). Therefore, a key element in IWM is prevention. Prevention aims to avoid critical situations in the field, by means of a wide range of cultural, mechanical, chemical, and biological control methods (Barzman et al., 2015; Oerke, 2006). Consequently, the deliberate maintenance of certain non‐crop plant populations in‐crop may lead to challenges for several objectives of weed management and agronomic practices that contribute to IWM. Furthermore, concerning the practical implementation of the threshold for weeds within sustainable crop production, the complexity of control measures, as well as educational requirements, monitoring efforts, and profitability, needs to be further considered (Bürger et al., 2008).

**Figure 1:**
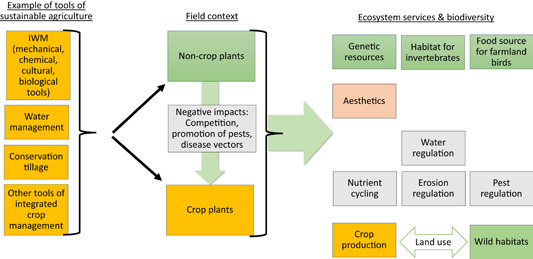
Interaction to be considered to ensure that protection goals are consistent with the requirements of sustainable agriculture for the delivery of the ecosystem service crop production. Interaction between good agricultural practices, ecosystem services, and disservices to be considered for defining protection goals. Provisioning services and required agricultural tools are in orange. Supporting and regulating services, including disservices, are in grey. Services and aspects related to biodiversity are in green. Cultural services are in red.

In summary, many different tools are required to support the ecosystem service crop production. Considering the populations of non‐crop plants in‐field, many of the tools and measures indicated above and used in IWM are not necessarily selective and have a broad spectrum of environmental consequences affecting both relevant ecosystem services and biodiversity (Figure 1). Mechanical tools in particular have numerous environmental consequences and trade‐offs to be considered, such as different forms of tillage (Norsworthy et al., 2012). These mechanical interventions interact not only with the ecosystem service crop production but also directly with supporting and regulating ecosystem services, such as water and erosion regulation or nutrient cycling. The environmental interactions can relate to soil degradation, erosion and compaction, microbial activity, carbon storage, leaching, fuel use, drainage, soil stability, and even availability of crop choices (Peigné et al., 2007). Local soil and site conditions are important to understand specific environmental consequences, which require tailored solutions and, at the same time, a high standard of management concerning those mechanical tools. Consequently, interactions can be both negative and positive depending on the specific field context, and they require flexible management decisions by the farmer to ensure a sustainable outcome.

Finally, to ensure that potential protection goals for non‐crop plants in‐field are met, the effects caused not only by mechanical tools but by any other and additional management practice that is part of GAPs must also be considered. Concerning in‐field protection goal options for NTTP, additional GAPs (e.g., concerning fertilization, crop rotations, and crop competitiveness) require detailed assessment, which also includes their common and acceptable environmental consequences. Especially with IPM, an ecosystem‐based strategy has been legally required in the EU since 2014, with a focus on the long‐term prevention of pests or their damage (Strand, 2000). IPM applies numerous techniques, ranging from biological control to habitat manipulation and use of pesticides. All of those agricultural tools that are used may potentially impact the population of non‐crop plants in the in‐field area.

As a result, the acceptable environmental impact and protection goal for an agricultural system, which should deliver the ecosystem service crop production, cannot be defined by focusing on a single tool, but on the combination of tools applied to a specific field context during a defined period. Here, integrated crop management considers simultaneously supporting and regulating services, GAPs, and ecosystem disservices (e.g., pests, diseases) to safeguard a successful and sustainable delivery of the ecosystem service crop production. Taking the example of IWM as indicated above, each of the non‐chemical tools that are used within the context to control weeds also exerts a range of specific impacts on the environment, and the risk assessment must account for those. Limiting assessments to chemical products only may therefore not be sufficient to ensure a SPG option to be achieved.

## TRADE‐OFFS BETWEEN ECOSYSTEM SERVICES AND BIODIVERSITY

With the different SPG options that are considered (Arts et al., 2017), diverging priorities for specific ecosystem services and biodiversity are underlie assumptions. The ecosystem service crop production was considered of primary importance in one of the options, however, without the requirements for its successful delivery in the context of GAPs and sustainable agriculture. Similarly, the other protection goal options do not consider their trade‐offs and relation to sustainable agriculture, and prioritize provision of habitat to invertebrates, food for farmland birds, and the protection of weeds of conservation concern over sustainable agriculture.

Generally, not all ecosystem services can be expected to be optimally available at the same time at the same location, and this is also the case for biodiversity (Holt et al., 2016), which relies on suitable availability and quality of habitats. Focusing on diverging in‐field ecosystem services for the same applied context, the protection goal options require an additional assessment of synergies and trade‐offs between the relevant ecosystem services (e.g., crop production, climate regulation, and aesthetic values) and corresponding weighing and priority setting (EFSA, 2016; Lele et al., 2013). A further assessment is also important in relation to specific habitat requirements to inform consistent biodiversity conservation. Focusing on biodiversity, agricultural production has always had multiple implications concerning habitats (Tscharntke et al., 2005). Those consequences range from the direct impact in the field, the adjacent environment to the implications of land use change, and corresponding loss of original habitats for biodiversity (Balmford et al., 2012; Clark & Tilman, 2017).

As an example for non‐crop plants in‐field, a persistent seedbank in the soil is a major challenge from an integrated agricultural production perspective (Barzman et al., 2015). Indeed, a reduced or depleted seedbank can allow a reduction in the amount of herbicides applied in IWM practices during subsequent years (Zhang et al., 2021). From the environmental perspective, however, the seedbank is considered an important component of vegetation dynamics and regarded as a reserve of biodiversity when NTTP are considered (EFSA, 2014). Although seedbank reduction is targeted in weed management (Norsworthy et al., 2012), this is considered a potential issue from an ecological perspective (EFSA, 2014), resulting in a major trade‐off requiring societal and technical discussion and decision making.

As another example, several weeds in agro‐ecosystems may be considered important from a nature conservation perspective (Marshall et al., 2003; Petit et al., 2010; Storkey & Westbury, 2007). Some weed species might provide food for predatory arthropod species also involved in biological pest control, thereby qualitatively supporting or modulating the ecosystem service “pest control.” However, weed species may also simultaneously act as reservoirs for vectoring insects or plant pathogens and thereby significantly promote pests and diseases, qualifying as further ecosystem disservice, which might require additional control measures (Lavina et al., 1996; Mantle & Shaw, 1977; Petit et al., 2010; Wisler & Norris, 2005).

Finally, to ensure consistent biodiversity conservation, both the availability and the quality of habitats must be considered (Robinson & Sutherland, 2002). For agricultural land use, a major question is how far is habitat quality compromised by a specific agricultural technique and which alternatives will be used by the farmer (with their own trade‐offs, for example, on habitat availability when crop rotations are changed). As a further example, different tools such as tillage and herbicide result in different characteristics of weed vegetation, its species composition, and corresponding agronomic challenges (Koning et al., 2019). This is a decisive component when indirect effects are suggested to be considered in risk assessments (EFSA, 2014).

Indeed, there are modern agricultural techniques that actively support biodiversity in‐field, such as conservation tillage within conservation agriculture, for example, owing to cover crops and crop rotation (Al‐Kaisi & Lal, 2017), or crop residues and weed seeds as food for insects, birds, and small mammals (Holland, 2004). Conservation tillage is a strategy that ensures delivery of vital ecosystem services, such as soil health by preventing soil erosion, improvement in soil structure and soil biota while contributing to global climate targets resulting from lower energy consumption and increased carbon sequestration (Holland, 2004). However, this practice also usually relies on the use of herbicides to ensure a sufficient and timely control of non‐crop plants (Al‐Kaisi & Lal, 2017). This demonstrates how synergies between specific agricultural practices, ecosystem services, and biodiversity can be achieved that are not currently supported in the protection goal framework.

On the other hand, considering the relationship between land use, agricultural activities, and biodiversity from a broader perspective, agricultural intensification also enables high‐yield farming, which provides new opportunities for landscape management solutions in favor of wild species by sparing remaining natural habitats (Balmford et al., 2018). Owing to competition between crops and weeds in‐field, any protection goal option with the aim of maintaining or tolerating a fixed amount of weed in‐field pushes farmers towards extensification of their crop production because weeds in‐field are responsible for major losses and increase the agronomic gap (Delmotte et al., 2011; Oerke, 2006; Tamene et al., 2016). This will result, in many cases, in a temporal increase in biodiversity for the field area, owing to the number of emerging weeds. It is questionable, however, whether the emerging weeds match those of conservation concern, which often require specific historical agricultural regimes to establish viable populations (e.g., missing seed purification steps for *Agrostemma githago*, compare Geisbauer & Hampicke, 2012).

Focusing on the delivery potential of desirable ecosystem services beyond crop production and biodiversity contributions from non‐crop plants in‐field, the impact and the environmental consequences of GAPs applied in a specific cropping system require a holistic assessment (Figure 1). Trade‐offs between the desired benefits and inherent hazards of complementary tools need to be identified and dealt with in a comprehensive risk assessment to ensure a realistic assessment frame about the risks at stake. Finally, contributions of the crop plants to ecosystem service delivery also need to be considered in this discussion, because they could also contribute, for example, to erosion control.

## CONCLUSION

To ensure that SPG for plant protection products are operational, it is important to consider the context of their use. When the context refers to the delivery of the ecosystem service crop production within sustainable agriculture, then the available toolkit of GAPs and their environmental interactions must be considered. Within the agricultural context, the in‐field weed community depends largely on the crop and cropping system and quickly adapts to the type of weed control applied (Koning et al., 2019). Therefore, IWM relies on diverse weed control tools that explicitly include chemical and non‐chemical methods and strategies. Furthermore, it is the availability of diverse tools and corresponding management methods that can also create opportunities for new habitats and biodiversity, for example, via conservation tillage.

However, as soon as diverging or even competing ecosystem services and biodiversity are considered within the same agricultural production, context trade‐offs can occur, which require a holistic assessment. Especially sustainable agriculture needs not only account for the environmental dimension but also the social and economic dimensions (Purvis et al., 2019). The way SPGs for environmental risk assessments are set can influence the ability of sustainable agriculture to adapt when facing new challenges and risks as they arise, for example, from a changing climate (Strand, 2000). The acceptance and applicability of protection goals require, therefore, a broad understanding of farming practices, the tools a farmer uses, and the effects of each tool on in‐field habitat availability and quality. Proactively considering the components of integrated crop management can, therefore, lead to a more functional system, balancing food, feed, and fiber production with environmental protection, and a consideration of the objectives of nature conservation. This will require additional interdisciplinary scientific research and development of approaches not just to understand but to design viable links between the farming community, consumers, researchers, and regulators.

## DISCLAIMER

The authors are employed at their corresponding companies Bayer AG, BASF SE, and Syngenta, respectively, as mentioned in the affiliations.

## Data Availability

There are no further data than the referenced citations in the article.
